# Effects of *Lycium barbarum* Polysaccharides on Immunity and the Gut Microbiota in Cyclophosphamide-Induced Immunosuppressed Mice

**DOI:** 10.3389/fmicb.2021.701566

**Published:** 2021-08-06

**Authors:** Ying Wang, Mingyi Sun, Hongyu Jin, Jianbo Yang, Shuai Kang, Yue Liu, Shuang Yang, Shuangcheng Ma, Jian Ni

**Affiliations:** ^1^Institute for Control of Chinese Traditional Medicine and Ethnic Medicine, National Institutes for Food and Drug Control, Beijing, China; ^2^School of Chinese Materia Medica, Beijing University of Chinese Medicine, Beijing, China; ^3^School of Medicine and Pharmacy, Ocean University of China, Qingdao, China

**Keywords:** *Lycium barbarum*, polysaccharides, immunity, gut microbiota, Caco2 cells

## Abstract

The mechanism of immunoregulation by *Lycium barbarum* polysaccharides (LBPs) was assessed by studying the effect of LBP on the immunity and the gut microbiota. LBP isolated and purified in this study was composed of nine monosaccharides, with an *M*w 1,207 kDa. LBP showed immunomodulatory activity in cyclophosphamide (Cy)-treated mice by restoring the damaged immune organs and adjusting the T lymphocyte subsets. We also found that LBP increased the diversity of the gut microbiota and the relative abundances of bacteria, such as *Rickenellaceae*, *Prevotellaceae*, *Bifidobacteriaceae*, and so on, which were positively associated with immune traits. In addition, Caco2 cells model was used to explore the intestinal absorption of LBP. Results showed that LBP was hardly absorbed in the intestine, which suggesting that most LBP may interact with gut microbiota. These findings suggest that the immune response induced by LBP is associated with the regulation of the gut microbiota.

## Introduction

The human gut microbiome encompasses >100 trillion microorganisms (Frank and Pace, 2008). About 10^13^–10^14^ microbial organisms are found in the gut of a healthy adult, far exceeding the total amounts of human cells (Muñoz-Garach et al., 2006). Gut microbiota disruption is increasingly described in multiple pathologies. Plant polysaccharides, which are usually indigestible components for the host, exert regulatory effects on the gut microbiota (Wang et al., 2019a). Recently, mounting evidence suggest that plant polysaccharides have obvious regulatory effects on the intestinal flora. Indeed, many studies have demonstrated that polysaccharides can improve the flora during disorders, restoring it to the normal state. For example, the polysaccharides of *Dendrobium Sonia* (Liu et al., 2019), *Hericium erinaceus* (Shao et al., 2019), and *Auricularia auricula* (Kong et al., 2020) are beneficial for gut microbiota composition.

Studies also demonstrated that intestinal microorganisms have critical functions in immune system development and maintenance by triggering immune reactions (Liu et al., 2018). For example, the gut microbiome upregulates major histocompatibility complex II (MHC II) and co-stimulatory molecules on antigen-presenting cells (APCs) increasing the amounts of cell surface molecules and cytokines (Tsuda et al., 2007). Short-chain fatty acids (SCFAs), the major metabolic products of polysaccharides in the intestine, have been attributed an important role in modulating multiple aspects of immunity. SCFAs affect both T cells and dendritic cells that induce immune responses (Belkaid and Hand, 2014). Reports indicating a strong correlation between the immune function of plant polysaccharides and the regulation of the intestinal flora shed some light on the mechanisms by which polysaccharides affect immune reactions.

The popular Chinese plant *Lycium barbarum* L. is widely utilized as food and in traditional Chinese medicine. Polysaccharides constitute the major bioactive ingredients of *L. barbarum*, with multiple effects, such as immunomodulatory, antioxidant, anti-hypertensive, and anti-tumoral activities (Gan et al., 2004). According to previous reports, *L. barbarum* polysaccharides (LBP) have good immunomodulatory effect (Zhang et al., 2014; Xie et al., 2017), and regulate the function of the gut flora (Zhou et al., 2018; Zhu et al., 2019). However, the association of LBP’s regulatory effects on the intestinal flora with its immune function remains largely unknown. A few research partly explained that the immune regulation of crude polysaccharide from *L. barbarum* may be related to the regulation of intestinal microflora by detecting the SCFA production in cecal content (Ding et al., 2019). In this study, to further demonstrate the immune enhancement mechanisms of LBP, the LBP was isolated and purified, its effects on immune function (immune organ index, immunoglobulin, and T lymphocyte subsets) and gut microbiota composition were assessed in the cyclophosphamide (Cy)-induced immunosuppressed mouse model. Meanwhile, the absorption of polysaccharides in the gastrointestinal tract is a key step in the mechanistic study of their action. In order to examine the role of LBP in the regulation of the intestinal flora and immune response, LBP transport was measured across Caco2 cells to elucidate the mechanism underpinning its intestinal absorption.

## Materials and Methods

### Materials

*Lycium barbarum* were collected from ZhongWei (Ningxia, China). The fruits of *L. barbarum* were confirmed by Professor NanPing Zhang from the National Institutes for Food and Drug Control (NIFDC), China. Bovine serum albumin (BSA) was provided by Thermo Fisher Scientific (Waltham, MA, United States). Cyclophosphamide (Cy), fluorescein isothiocyanate (FITC), and Hank’s balanced salt solution (HBSS) were purchased by Sigma-Aldrich (Germany). D-glucose (Glc), D-Mannose (Man), L-arabinose (Ara), D-galactose (Gal), L-rhamnose (Rha), D-ribose (Rib), D-xylose (Xyl), D-glucuronic acid (GlcA), and D-galacturonic acid (GalA) were obtained from the NIFDC. FITC labeled CD4 (100406), phycoerythrin (PE) labeled CD8 (250304), and allophycocyanin labeled CD3 (100235) anti-mouse monoclonal antibodies were obtained from BioLegend (San Diego, CA, United States). Toyopearl HW-65F resins (30~60 μm) were purchased from TSK corporation, which is used in size exclusion chromatography.

### LBP Purification

The powder of *L. barbarum* (30.0 g) was defatted using ether for 2 h, and a rotary evaporator was utilized for concentration. Next, the samples were treated with 80% ethanol for 1 h (twice) for small-molecule material and pigment removal. This was followed by two water extractions at 80°C for 1 h (Redgwell et al., 2011). The aqueous phase was filtered, and submitted to precipitation for 24 h with ethanol until alcohol levels reached 80% at ambient. The resulting precipitate underwent lyophilization to yield crude polysaccharides, with protein removal by the Sevag’s technique. These crude polysaccharides were sequentially isolated by size-exclusion and anion exchange chromatography. First, crude polysaccharides were dissolved in ultra-pure water and eluted with NaCl solutions at 0.1, 0.2, and 0.5 M, respectively, supplied at 5 ml/min (Zhu et al., 2013). The fractions were assessed for carbohydrate amounts by the phenol-sulfuric acid assay. The main fractions further underwent fractionation by size-exclusion chromatography on a HW-65F column (26 mm × 600 mm), with 0.1 M NaCl as eluent. The total carbohydrate contents of various fractions were monitored by the phenol-sulfuric acid method. Then, the major fractions were obtained and submitted to dialysis and lyophilization to yield the purified polysaccharides (550 mg).

### Analytical Methods for Composition Determination

#### Determination of the Total Carbohydrate Content

Neutral sugar (D-glucose equivalents) and uronic acid (D-galacturonic acid as reference) amounts were assessed by the phenol-sulfuric acid and sulfuric acid-carbazole methods, respectively (Liu et al., 2015).

#### Molecular Weight Assessment

*Lycium barbarum* polysaccharides *M*w was determined on a high performance liquid chromatography (HPLC) instrument (Shimadzu, Japan), comprising a refractive index detector (RID) and a multi-angle laser light scattering detector (MALLS, DAWN HELEOS, Wyatt Technology, United States). The samples (10 mg) in 2 ml of the eluent underwent filtration with a 0.45 μm membrane (Wu et al., 2016). Several size-exclusion columns, including Shodex SB806 (300 mm × 7.8 mm, i.d.) and Shodex SB804 (300 mm × 7.5 mm, i.d.), were utilized for good resolution. Elution was carried out with 0.1% NaCl at 0.5 ml/min. An injection volume of 50 μl was used, and samples were run for 60 min at 40°C.

#### Monosaccharide Composition Analysis

*Lycium barbarum* polysaccharide samples (1 mg/ml) were treated with trifluoroacetic acid (TFA, 4 M) at 120°C for 4 h, and complexed with PMP (0.5 M; Wang et al., 2019b). Then, 10 μl of the resulting solution was assessed by HPLC-PDA on a ZORBAX Eclipse XDB-C_18_ column (250 × 4.6 mm, 5 μm; Agilent, United States) with UV monitoring at 250 nm. Acetonitrile and 0.125 M KH_2_PO_4_ (4:21 v/v; pH 6.9) were employed for elution at 1.0 ml/min.

### Measurement of Immunomodulatory Activity

Female Kunming mice (6 weeks old, 28–32 g) underwent a 1-week acclimatization in cages (10/cage) with rodent chow [Certificate of Conformity: SCXK (ShangHai) 2008–0016] and water at will, under a 12/12 h light-dark cycle at 22 ± 2°C with 55 ± 5% humidity. All procedures concerning animal experiments were conducted following the “Guide for the Care and Use of Laboratory Animals” published by the National Institutes of Health and approved by the Experimental Animal Ethics Committee of the Academic Committee of Beijing University of Chinese Medicine.

#### Treatments and Study Design

Upon acclimatization, the animals were randomized to the control, model (Cy), low (Cy + low dose LBP) and high (Cy + high dose LBP) dose LBP, and the positive control (Cy + lentinan) groups (*n* = 8/group). Control animals underwent i.p. administration of normal saline, while the remaining animals were administered 100 mg·kg^−1^ of Cy for 3 days to achieve immunosuppression. Upon modeling, the control and model groups were i.g. administered 0.5 ml of sterile water; the positive control group was injected with lentinan at 10 mg·kg^−1^, while the Cy + low and Cy + high dose LBP groups were i.g. administered with 50 and 100 mg·kg^−1^ LBP (0.5 ml), respectively. The doses of LBP were based on the previous study (Ding et al., 2019). All animals received 11 consecutive daily i.g. treatments. On the last day, the fecal samples of all mice were obtained and stored at −80°C until further analysis.

At sacrifice, the spleen and thymus were weighted to assess organ indices: Organ index = organ weight (mg)/body weight (g).

#### Preparation of Mouse Splenocytes

Spleens collected from Kunming mice under aseptic conditions, placed in cold phosphate buffered saline (PBS) and filtration through a 200-mesh sieve to obtain single-cell suspension. The splenocytes were collected by centrifugation at 4,000 rpm for 20 min, and resuspended in erythrocyte lysis buffer for 5 min to remove the red blood cells. The erythrocytes were removed by centrifugation at 4,000 rpm for 20 min, and the splenocytes were washed and resuspended in RPMI-1640 medium (10% FBS; Li et al., 2017).

#### Serum IgG and IgM Measurements

After eye removal, venous blood specimens (1 ml) were obtained and submitted to centrifugation for serum preparation. IgG and IgM amounts were determined by ELISA using specific kits (Abcam Inc., England) as directed by the manufacturer.

#### CD4+ and CD8+ T Lymphocyte Assessment

Single-cell suspensions of splenocytes were submitted to incubation with mouse anti-CD4-FITC (5 μl), anti-CD8-PE (5 μl) and anti-CD3-APC antibodies, respectively, at 4°C for 1 h away from light. The FC 500 flow cytometry system (Beckman Coulter Inc., United States) was used for analysis, with CD4+ and CD8+ T lymphocyte amounts expressed as percentages of all T cells.

### Gut Microbiota Analysis

Genomic DNA extraction from fecal samples was performed with TIANamp Stool DNA Kit (Tiangen Biotech Co., China) as directed by the manufacturer. The V3–V4 region of bacterial 16S rRNA was amplified with Primers F (Illumina adapter sequence 1+ ACTCCTACGGRAGGCAGCAG) and R (Illumina adapter sequence 2+ GGACTACHVGGGTWTCTAAT). Genesky Biotechnologies (China) carried out sequencing and bioinformatics on an Illumina MiSeq platform, generating 2 × 250 bp paired-end reads. High-quality sequences were clustered with UPARSE according to Ribosomal Database Project (RDP) into operational taxonomic units (OTUs; 97% similarity cutoff). Finally, α- and β-diversities, as well as species screening were performed according to the abundance levels of OTUs with the R package.

### Transport of LBP Across Caco2 Cells

#### Preparation of Fluorescently Labeled LBP

One hundred milligrams of LBP were added to 10 ml sodium carbonate buffer (0.5 M, pH 8.0) containing 25 mg FITC (Min et al., 2015). Upon incubation for 24 h at ambient away from light, the samples were centrifuged (4,500 rpm/min, 10 min), and the resulting supernatants underwent precipitation within 24 h with ethanol until alcohol levels reached 80% at ambient. The precipitates were dissolved in 10 ml hot water and then precipitated with absolute ethanol again. The obtained precipitates were repeatedly washed with ethanol and freeze-dried to obtain FITC-LBP.

#### Cell Culture

The Caco2 cell line was purchased from the national infrastructure of cell line resources (China), and cultured in MEM containing 20% FBS, 1% NEAA, and 1% penicillin and streptomycin. The cells were incubated at 37°C in a humid atmosphere with 5% CO_2_. At 80–90% confluence, subculture was performed upon treatment with 0.25% trypsin. Cells were used between passages 30 and 40 in this study.

#### FITC-LBP Transport Through the Caco2 Cell Monolayer

Caco2 cells were plated at 2 × 10^5^ cells/ml on polyester membranes in transwell chambers underwent incubation for 21 days to generate a monolayer, whose integrity was assessed at 2–3 day intervals by transepithelial electrical resistance (TEER) measurement on a Millicell-ERS (Millipore, Unites States), after three washes with HBSS at 37°C.

Fluorescein isothiocyanate-*Lycium barbarum* polysaccharide transfer from the apical (AP) side to the basolateral (BL) one was monitored with 0.5 ml of FITC-LBP solution and 1.5 ml of HBSS added to the AP and BL sides, respectively. At set times, removal of 600 μl of solution from the BL side was performed, followed by immediate addition of 600 μl HBSS. FITC-LBP transfer from BL side to the apical one was assessed with 1.5 ml of FITC-LBP solution and 0.5 ml of HBSS added to the BL and AP sides, respectively. At set times; removal of 200 μl of solution from the AP side was performed, with immediate supplementation of 200 μl of HBSS.

Next, the content of FITC-LBP was measured by a HPLC method, on a Waters e2695 series system (Waters Corporation, United States) fitted with a Shodex SB805 (300 mm × 7.5 mm, i.d.) column and a fluorescence detector (FLD). HBSS was utilized for elution at 0.5 ml/min at 30°C, and fluorescent signals were acquired at 485-nm excitation and 515-nm emission. A total of 10 μl of the sample solution was injected for each run. The R (transfer rate) of FITC-LBP was determined by FITC-LBP amounts at BL and AP sides at various times. The *Papp* (apparent permeability coefficient) was obtained as follows: *Papp* = (dQ/dt)/(A × C_0_), where A represents the surface of the transwell plate (1.12 cm^2^), dQ/dt represents FITC-LBP amounts/unit time in the transwell plate, and C_0_ represents the initial FITC-LBP levels in the donor chamber.

### Statistical Analysis

Data are presented as mean ± SD, and were compared by one-way ANOVA. *p* < 0.05 indicated statistical significance.

## Results

### LBP Composition

Neutral sugar and uronic acid contents were assessed and the results showed that LBP contained carbohydrates and uronic acids at 73.2 ± 3.7 and 18.5 ± 2.5%, respectively. The monosaccharides of LBP were analyzed by the HPLC-PDA technique. The results indicated that LBP was composed of Man, Rib, Rha, GlcA, GalA, Glc, Gal, Xyl, and Ara. Table 1 shows the molar ratio of nine types of monosaccharides in LBP.

**Table 1:** Monosaccharides of LBP.

	Man	Rib	Rha	GlcA	GalA	Glc	Gal	Xyl	Ara
Mol%	3.5	3.3	3.8	1.8	22.2	11.0	20.8	3.7	29.9

**Figure 1:**
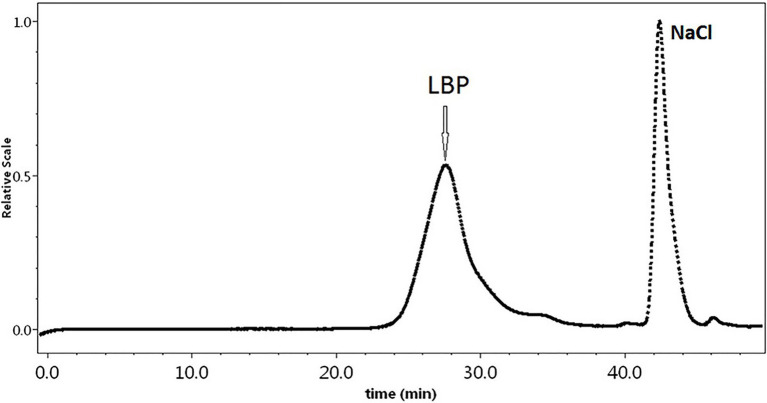
The HPGPC chromatograms of *Lycium barbarum* polysaccharide (LBP) samples.

The *M*w of LBP was determined by the HPGPC-RI-MALLS method, which does not rely on column calibration standards or elution features (Hu et al., 2013). We found an *M*w of 1,207 kDa for LBP (Figure 1). The polydispersity index of this polysaccharide fraction was 1.43, suggesting that LBP obtained in our study is a relatively homogeneous component.

### LBP Effects on Immunosuppressed Mice

**Figure 2:**
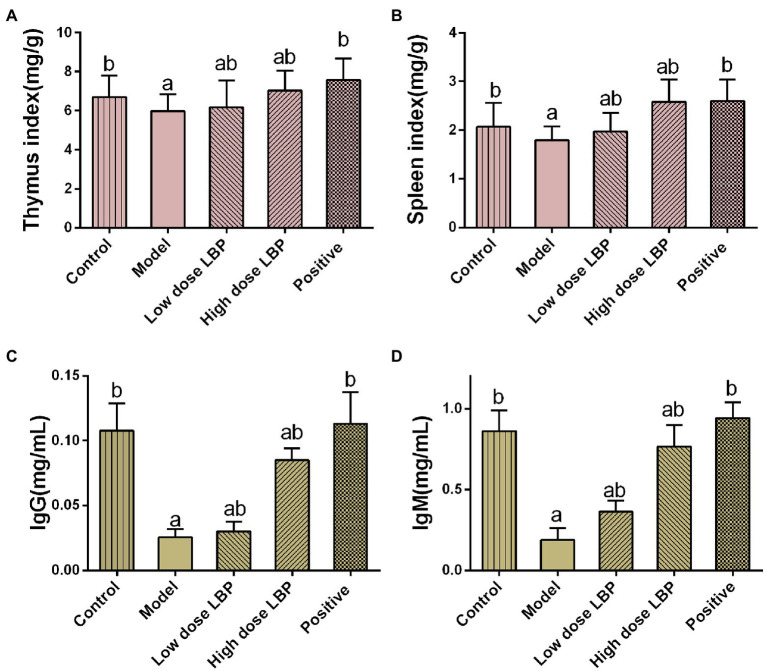
The immunomodulatory effects of LBP in cyclophosphamide (Cy)-treated mice. **(A)** Spleen index, **(B)** thymus index, **(C)** IgG amounts, and **(D)** IgM amounts. Values are mean ± SD (*n* = 8). ^a^*p* < 0.05 vs. control group; ^b^*p* < 0.05 vs. model group.

To evaluate the potential immunomodulatory effects of LBP, spleen and thymus indices were assessed. As depicted in Figure 2, spleen and thymus indices in the model group were markedly reduced compared with control values (*p* < 0.05). Upon administration of low-dose and high-dose LBP groups, the thymus index was elevated by 3.34 and 17.7%, respectively, in comparison with the model group. Similarly, the spleen index was 9.44 and 43.3% higher in the low-dose and high-dose LBP groups compared with the model group, respectively. These findings suggested that LBP alleviated Cy-associated spleen and thymus atrophy. To assess LBP effects on humoral immunity in Cy-treated mice, serum IgM and IgG levels were evaluated by ELISA. As depicted in Figure 2, serum IgG and IgM amounts in the Cy group had significant reductions in comparison with control values (*p* < 0.01). Meanwhile, serum IgG and IgM amounts in LBP pretreated groups were starkly increased in comparison with those of the model group (*p* < 0.05). Specifically, in the 80 mg/kg/d LBP group, IgG and IgM amounts almost returned to normal, but lower than the positive control group. The above findings suggested that LBP improved humoral immunity following Cy treatment.

**Figure 3:**
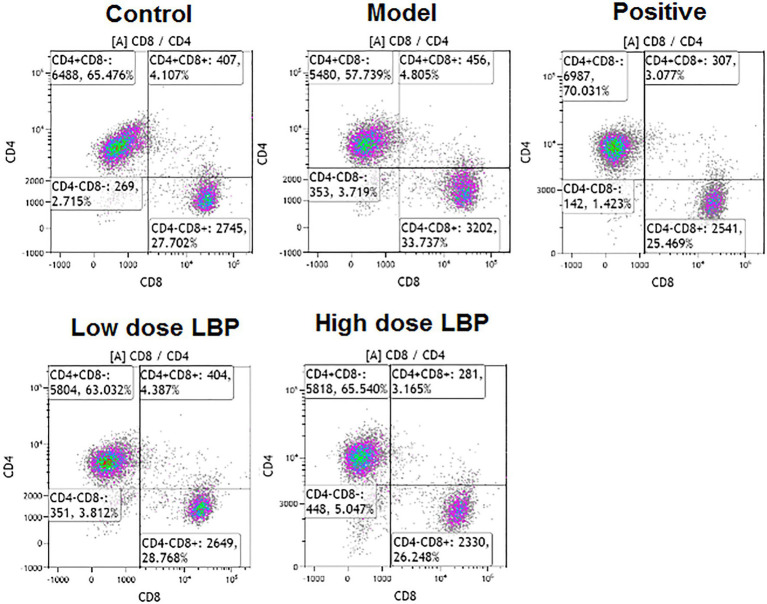
Representative flow-cytograms depicting of CD4+ and CD8+ T cell rates among T cells in immunosuppressed mice.

**Figure 4:**
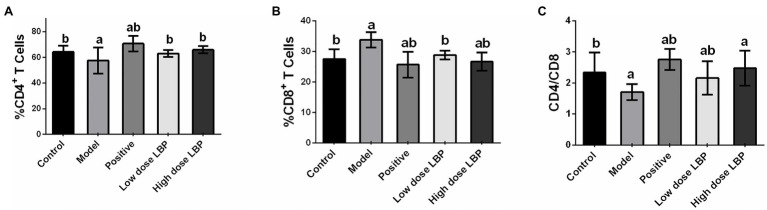
Splenic CD4+ and CD8+ T cell rates in immunosuppressed mice. **(A–C)** Statistical analysis of the percentages of CD4+ and CD8+ T cells and CD4+/CD8+ ratios. Data are mean ± SD (*n* = 8). ^a^*p* < 0.05 vs. control group; ^b^*p* < 0.05 vs. model group.

In this study, splenic CD4+ and CD8+ T cell rates were assessed flow-cytometrically, as shown in Figure 3. The low-dose and high-dose LBP increased CD4+ T cell amounts to 63.06 and 65.99%, respectively, compared with the Cy group (57.72%), suggesting that LBP reversed the Cy-induced reduction of CD4+ T cells (Figure 4). We also found that CD4+/CD8+ amounts in the Cy group were markedly decreased compared with control values. However, after treatment with low-dose and high-dose LBP, CD4+/CD8+ rates were 2.19 and 2.48, respectively, i.e., markedly increased compared with the Cy group (1.71). In previously reports, elevated CD4+/CD8+ rate reflects a better status of overall immunity (Wang et al., 2020). These results indicated that the immune function of the host could be upregulated by LBP.

### LBP’s Effects on the Gut Microbiota

**Figure 5:**
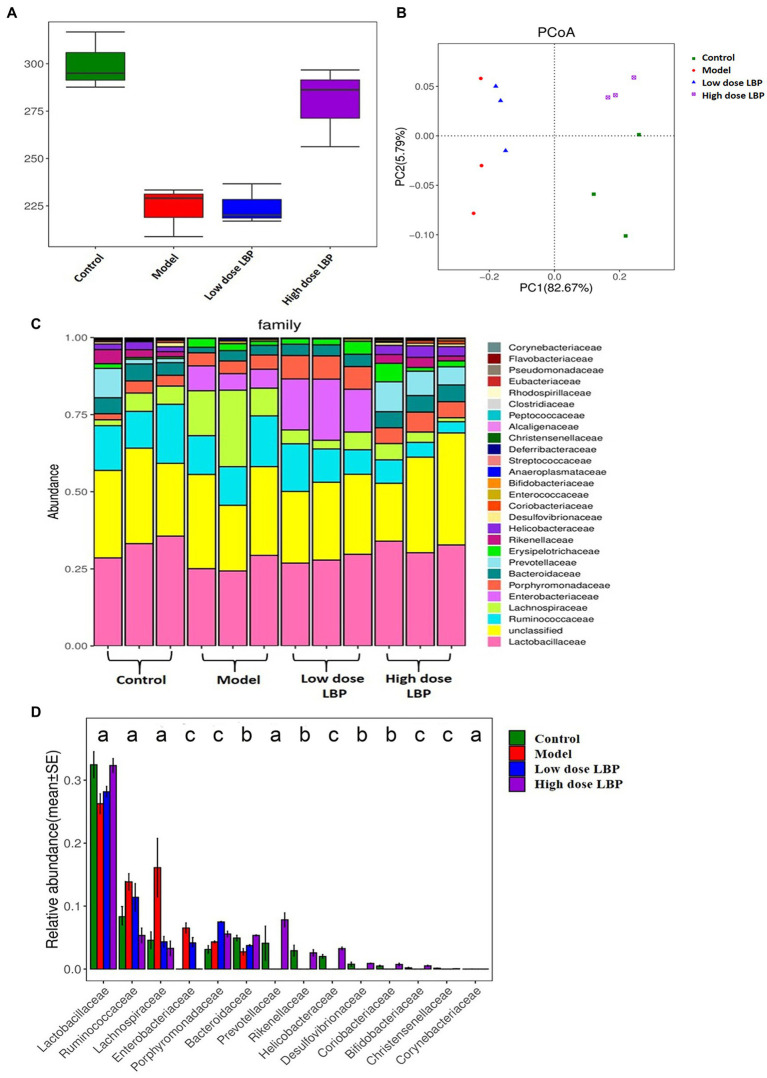
*Lycium barbarum* polysaccharide’s effects on fecal microbiome. Data are presented as mean ± SD (*n* = 3). **(A)** Fecal taxon richness evaluated by α-diversity analysis based on the ace technique. **(B)** β-diversity (Unifrac) of fecal microorganisms, based on principal coordinates analysis (PCoA) of the Unifrac distance matrices. Every dot represents a specimen, and groups are distinguished by colors (green, control; orange, model; blue, low dose LBP; and purple, high dose LBP). **(C)** Microbiome taxon composition at the family level. **(D)** Analysis of the differences among the experimental groups based on gut microbiota composition at the family level (^a^*p* < 0.05, ^b^*p* < 0.01, and ^c^*p* < 0.001).

The bacterial 16S rRNA V3-V4 region was sequenced in fecal samples, revealing a high microbial diversity with totally 52,534 sequences, representing 530 OTUs. To determine whether fecal taxon richness is affected by LBP, α-diversity analysis was carried out by the ACE method (Figure 5A), the control and model groups had marked differences, indicating that Cy decreased taxon richness in these specimens. Compared with the model group (227.8 ± 22.4), taxon richness in fecal samples from mice treated with high-dose LBP (284.8 ± 30.2) was increased significantly, and closer to that of the normal control group (292.8 ± 20.2).

Next, principal coordinates analysis (PCoA) data (Figure 5B) indicated that the control and model groups were overtly separated. Expectedly, LBP overtly affected gut microbiota composition. The first two principal components (PC1 and PC2) were assessed for various samples. On PC1 explaining 82.67% of the total variance, the control and LBP_high dose groups overlapped, suggesting that high dose LBP shifted the gut microbiota in model animals toward control composition. However, the low dose LBP and model groups were not significant different.

At the phylum level, the LBP group mostly had three phyla, including *Firmicutes*, *Bacteroidetes*, and *Proteobacteria*, making up about 95% of all microorganisms. However, a higher relative abundance of *Firmicutes* was observed after oral administration of high dose LBP. As displayed in Figures 5C,D, it was found that 14 major families among experimental groups showed significant difference. After Cy administration, markedly decreased relative abundance rates of *Rikenellaceae*, *Bacteroidaceae*, *Prevotellaceae*, *Lactobacillaceae*, *Bifidobacteriaceae*, *Christensenellaceae*, *Coriobacteriaceae*, *Desulfovibrionaceae*, *Porphyromonadaceae*, and *Helicobacteraceae* were found, alongside significantly elevated relative abundance rates of *Lachnospiraceae*, *Ruminococcaceae*, *Enterobacteriaceae*, and *Corynebacteriaceae* in comparison with control amounts (Figure 5D). The results also showed that administration of high dose LBP overtly elevated the relative abundance rates of *Lactobacillaceae*, *Bacteroidaceae*, *Prevotellaceae*, and so on in comparison with the model group (*p* < 0.05). Moreover, LBP intervention markedly reversed Cy-induced elevations of the relative abundance rates of *Lachnospiraceae*, *Ruminococcaceae*, and *Enterobacteriaceae*.

**Figure 6:**
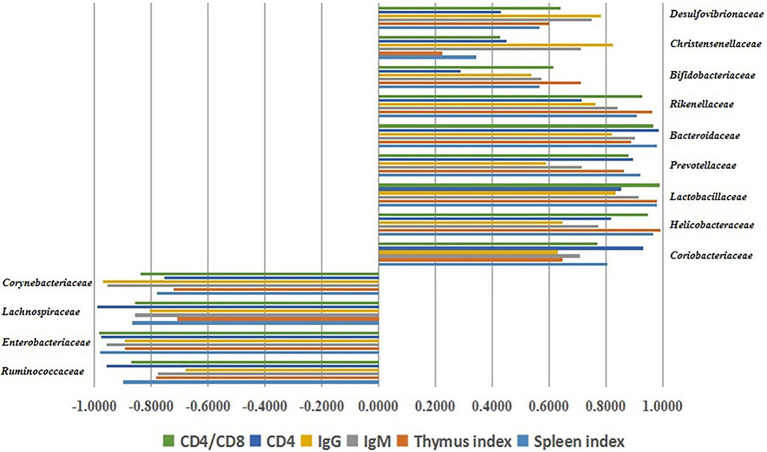
Associations of the gut microbiota constituents at the family level with immune traits, assessed by Pearson’s correlation analysis.

In order to assess the contribution of LBP to the regulation of the intestinal flora in the development of immunity, Pearson’s correlation analysis was performed to assess the relative abundance levels of the major gut microorganisms at the family level and immune function indexes. The correlations were expressed in *R* (Figure 6). Pearson analysis showed that the relative abundance rates of some intestinal microflora constituents were closely associated with immune organ index (spleen and thymus indexes), immunoglobulins (IgG and IgM), and CD4+ T cells. *Rickenellaceae*, *Bacteroidaceae*, *Prevotellaceae*, *Lactobacillaceae*, *Bifidobacteriaceae*, *Christensenellaceae*, *Coriobacteriaceae*, *Desulfovibrionaceae*, and *Helicobacteraceae* had significant positive correlations with immune traits (*p* < 0.05), while *Enterobacteriaceae*, *Lachnospiraceae*, *Corynebacteriaceae*, and *Ruminococcaceae* were negatively correlated with immune traits (*p* < 0.05). These findings indicated that the immune response induced by LBP is associated with gut microbiota regulation.

### LBP Cytotoxicity in Caco2 Cells

**Figure 7:**
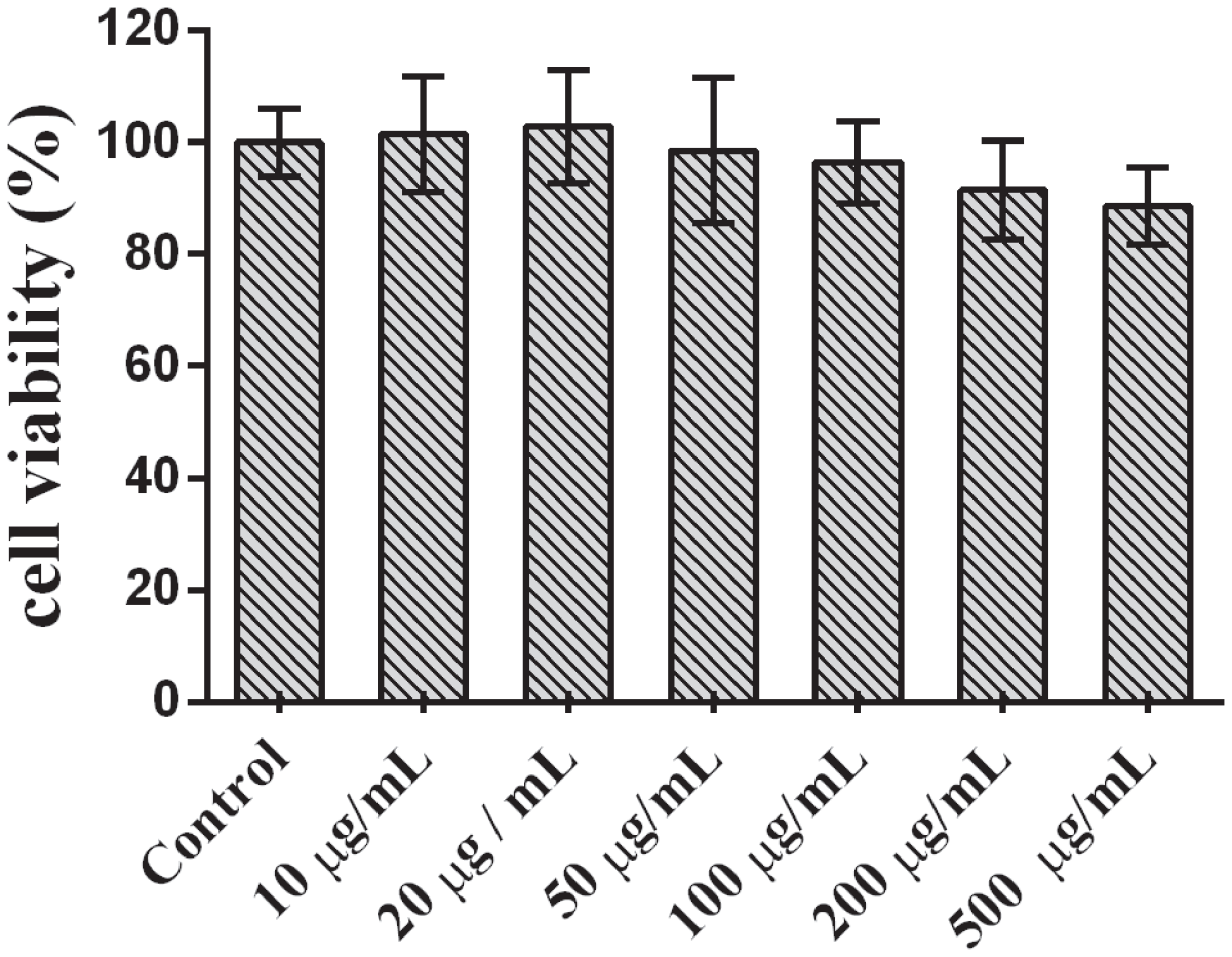
*Lycium barbarum* polysaccharide cytotoxicity in Caco2 cells.

*Lycium barbarum* polysaccharide cytotoxicity in Caco2 cells was determined by the MTT assay, and results are depicted in Figure 7. At LBP concentrations of 10–100 μg/ml, Caco2 cell viability was above 90%, suggesting that LBP was not overtly toxic to cells within this concentration range and could be used in the following experiments.

### Determination of Accumulative Transfer Amounts of LBP in Caco2 Cells

**Figure 8:**
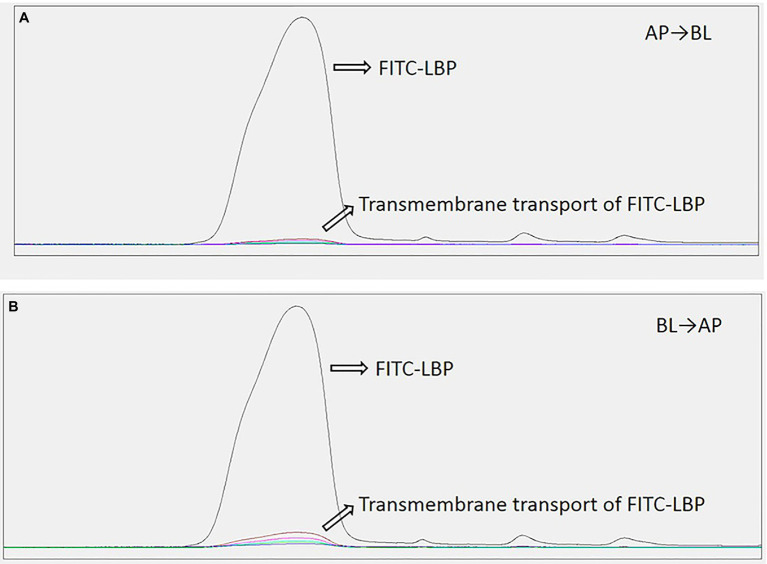
Transport levels of LBP in Caco2 cells administered 50 μg/ml LBP. **(A)** From apical (AP) to basolateral (BL) side. **(B)** From BL to AP side.

The R (transfer rate) of FITC-LBP was studied by using the Caco2 cell model. The R was obtained as follows: R = C_t_/C_0_, where C_t_ represents the cumulative transport amount, and C_0_ represents initial added amount. The samples solutions at the AP and BL sides collected at different time periods from 0 to 180 min were detected by HPLC-FLD. As shown in Figure 8, polysaccharide concentrations at the AP and BL sides increased with time, with a time-dependent relationship. These results indicated that LBP could produce a certain amount of transmembrane transport by prolonging incubation time. The R values of FITC-LBP within 180 min were 0.92 and 0.98%, respectively, from AP to BL and vice versa, which showed that the transmembrane transport of LBP was very limited. Meanwhile, the *Papp* value were 0.822 × 10^−6^ cm/s from AP to BL side, and 0.811 × 10^−6^ cm/s from BL to AP side.

## Discussion

In this study, the LBP was isolated and purified, its effects on immune function (immune organ index, immunoglobulin, and T lymphocyte subsets) and gut microbiota composition were assessed in the Cy-induced immunosuppressed mouse model. Cy treated mice were selected as the test model because Cy can induce immunosuppression (Xu and Zhang, 2015) and affect the gut microbiota (Tang et al., 2014), which is widely used in multiple recent reports. In the current assays, Cy increased the amounts of *Enterobacteriaceae* while decreasing *Bacteroidetes* levels, corroborating previous reports. Lentinan is often used as a positive control group because of its significant immunomodulatory effect in many reports. In this study, lentinan treated mice were also selected as positive control group.

The spleen and thymus represent critical immune organs. Immunopotentiators could increase thymus and spleen weights (Mei et al., 2013). Elevated organ indices were found in animals administered LBP, indicating that LBP could counter the Cy-associated immune organ atrophy. Generally, mature T cells include two main cell categories, i.e., CD4+ and CD8+ T cells. It is well-known that CD4+ and CD8+ are T helper (Th) and T cytotoxic (Tc) lymphocytes, respectively. CD4+ and CD8+ T cells and its ratio are considered as a vital index in immunocompetence evaluation. Many investigators have reported that the high ratio of CD4+/CD8+ would be favor of immunocompetence. And it has been proved that polysaccharides can modulate immunocompetence by adjust the levels of CD4+ and CD8+ T cells (Zuckermann, 1999; Wang et al., 2019c). In this work, the decreases of CD4+ T lymphocytes and CD4+/CD8+ ratio induced by Cy were reversed by treatment with LBP. Humoral immune response is also essential for adaptive immunity, which is triggered by specific antigens (Zhao et al., 2014). In this study, compared with the model group, LBP treatment resulted in significant increments in globulin contents, indicating that LBP improves the humoral immunity. The increase of the proportion of CD4 cells is also one of the factors leading to the increase of globulin level. It is well known that CD4+ T lymphocytes contain secrete-related factors, which improve the production of antibody and regulate the function of immunocompetence (Chen et al., 2016). In general, LBP regulates immunity by enhancing humoral and cellular immune pathways.

The gut microbiota are known to affect the immune system and gut homeostasis by triggering immune responses and maintaining barrier function in the epithelium. Polysaccharides alter bacterial composition and metabolites, which in turn modulate host immunity (Borghi et al., 2017). In this study, the effects of LBP on mouse gut microorganisms upon Cy treatment were evaluated. The results showed that LBP could raise OTU amounts and β-diversity in mice in comparison with the Cy group. It is admitted that reduced species numbers and diversity reflect altered immune function. It was found that *Rickenellaceae*, *Bacteroidaceae*, *Prevotellaceae*, *Lactobacillaceae*, *Bifidobacteriaceae*, *Christensenellaceae*, *Coriobacteriaceae*, *Desulfovibrionaceae*, and *Helicobacteraceae* had significant positive correlations with immune traits (*p* < 0.05). Previous studies have shown that *Prevotellaceae* are related to TGF-β3, which is a cytokine regulating intestinal barrier function (Million et al., 2018). *Rickenellaceae* are an important microflora group producing SCFA in the intestine. *Bifidobacteriaceae* in the LBP group indicated that SCFA production was increased (Zhang et al., 2017). The above results also showed that LBP remarkably reduced the amounts of *Escherichia coli* and *Enterobacteriaceae*, which is opportunistic pathogens in fecal specimens (Yang et al., 2013). At the genus level, LBP high-dose enhanced the abundance rates of *Lactobacillus*, *Bacteroides*, and *Bifidobacterium*. Many studies demonstrated that *Lactobacillus* and *Bifidobacterium* are healthy microbiota constituents that boost gut mucosal immunity. Furthermore, *Lactobacillus* induces immune reactions by triggering the secretion (Galdeano and Perdigon, 2006). According to published reports, *Bifidobacterium* enhances immune reactions by boosting interleukin 6 (IL-6) and IL-8 biosynthesis *via* NF-κB signaling (Turroni et al., 2014). Moreover, *Bifidobacterium* can improve epithelial cell barrier function and enhance gut immune response (Wang et al., 2014). *Bacteroides* could restore DCs’ ability to induce T-cell proliferation (Cano et al., 2012). In addition, we found that the low-dose LBP group which hardly achieved the restoration of the intestinal flora shows weak immunoregulation effect.

The balance of Th17 and Treg cells can help the improvement of diseases in many cases. The recent findings have showed that specific gut microbiota organisms and metabolites can affect the balance of Th17 and Treg cells (Cheng et al., 2019). It has been demonstrated that *Bacteroides* could interact with Treg to promote IL-10 production by producing metabolites (Luo et al., 2017). And Tan et al. (2016) proposed that one species of *Bifidobacteria* could induce Th17 cells in the murine intestine. The effect of LBP on immune function may be related to maintaining the balance of Th17 and Treg cells through gut microbiota, which needs to be further proved by measuring Treg and Th17 cells.

A prominent mechanism by which microbiota shape the immune response is *via* SCFAs, which are the major metabolites produced by the gut microbiota are not only energy substrates for the colonic epithelium, but also exert important effects on the immune system (Tremaroli and Backhed, 2012). It has been reported that fermentation ability depends upon the structural features of polysaccharides. Specifically, some polysaccharides containing structures such as arabinoxylan and arabinogalactan can be metabolized by the gut flora (Dion et al., 2016; Mendis et al., 2016); further affecting the immune system by microbiota-dependent mechanisms and/or through gut associated lymphoid tissue (GALT). The above results showed the molar ratio of arabinose, galactose, and xylose in LBP were 29.9, 20.8, and 3.7%. Meanwhile, previous studies have shown that arabinogalactan is the main structural region of LBP (Liu et al., 2016). Therefore, the effect on the intestinal flora of LBP may be closely related to its structure. Moreover, the SCFAs in cecal content will be detected in the next study to further explain the immunoregulation mechanism of LBP.

As we have obtained from our experimental data, LBP exhibit superior immunoregulation and can effectively modulate the composition of the gut microbiota (Zhou et al., 2018). However, the relationship between immune regulation and gut microbiota regulation of LBPs remains to be elucidated. The transport of LBP in the intestine is very important for elucidating the immune mechanism of polysaccharides. Most polysaccharides are not easily digested and degraded into monosaccharides or oligosaccharides under the action of gastric juice, but exist in the form of polysaccharides in the digestive tract. In addition, it is generally believed that most polysaccharides cannot be directly absorbed. A previous report on LBP metabolism *in vitro* showed that LBP with an *M*w of 1,720 kDa does not degrade in artificial gastric juice and small intestinal fluid, safely reaching the large intestine (Ding et al., 2018). In this study, Caco2 cells were tested as a cell model mimicking the human gut epithelium to assess LBP’s intestinal transport.

Cultured Caco2 cells, forming a confluent monolayer, are widely used in drug transport research. The TEER of the Caco2 cell monolayer was assessed to verify its integrality (Wang et al., 2018). The results suggested that the Caco2 cell monolayer model was established successfully. Moreover, in order to improve the sensitivity and specificity of LBP determination, LBP was first labeled with FITC. In the Caco2 cell transport experiment, FITC-LBP in samples was determined by the FLD detector, which could greatly improve the sensitivity of detection. In this study, the detection limit of FITC labeled LBP was 0.1 μg/ml. A previous study (Cai et al., 2017) showed that LBP time-dependently traverses the Caco2 cell monolayer. Based on the above experiments, the R and *Papp* values of FITC-LBP from AP to BL side were 0.92% and 0.822 × 10^−6^ cm/s, respectively, consistent with the previous study. However, the current results also indicated that the transmembrane transport of LBP was very small. The *Papp* of a drug traversing the Caco2 cell monolayer is well-correlated with its absorption in the body (Artursson and Karlsson, 1991). It is considered that at *Papp* < 1.0 × 10^−6^ cm/S, drug absorptivity is lower than 20%, while *Papp* > 10 × 10^−6^ cm/S reflects a drug absorptivity higher than 70%, indicating a well-absorbed drug. The above results, with *Papp* < 1.0 × 10^−6^ cm/s, suggested that LBP was hardly absorbed in the intestine.

## Conclusion

The mechanism of immunoregulation by LBP remains under exploration. In the present study, LBP was mainly composed of nine sugars, with an *M*w 1,207 kDa. LBP showed immunomodulatory activity in Cy-treated mice by restoring the damaged immune organs, and enhancing humoral and cellular immune pathways. We also found that LBP could raise the number and diversity of gut microorganisms, as well as the relative abundance levels of bacteria, such as *Rickenellaceae*, *Prevotellaceae*, *Bifidobacteriaceae*, and so on. In addition, it is also found that these bacteria had significant positive correlations with immune traits (*p* < 0.05). To explore the mechanism of immunoregulation by LBP, the intestinal absorption of LBP was studied in the Caco2 cell monolayer model. The results showed that the *Papp* of LBP was less than 1.0 × 10^−6^, suggesting that it is hardly absorbed in the intestine. These findings preliminarily indicate that the regulation by LBP of the intestinal microflora is an important step in immune regulation. The total SCFAs in the cecum, cytokines, and the type of CD4 cell (Th17, Treg) will be determined in next experiments to further explain the correlation between gut microbiota and immune function.

## Data Availability Statement

The original contributions presented in the study are included in the article/supplementary material, further inquiries can be directed to the corresponding authors.

## Ethics Statement

The animal study was reviewed and approved by Animal Experiment Ethics Committee of National Institutes for Food and Drug Control.

## Author Contributions

SM and JN: conception and design of study. YW, MS, HJ, and SK: acquisition and analysis of data. YW and SY: drafting of manuscript. JY and YL: revising of manuscript. All authors contributed to the article and approved the submitted version.

## Conflict of Interest

The authors declare that the research was conducted in the absence of any commercial or financial relationships that could be construed as a potential conflict of interest.

## Publisher’s Note

All claims expressed in this article are solely those of the authors and do not necessarily represent those of their affiliated organizations, or those of the publisher, the editors and the reviewers. Any product that may be evaluated in this article, or claim that may be made by its manufacturer, is not guaranteed or endorsed by the publisher.
